# Mechanisms of γδ T cell accumulation in visceral adipose tissue with aging

**DOI:** 10.3389/fragi.2023.1258836

**Published:** 2024-01-11

**Authors:** Sujata Mukherjee, Maria E. C. Bruno, Jason Oakes, Gregory S. Hawk, Arnold J. Stromberg, Donald A. Cohen, Marlene E. Starr

**Affiliations:** ^1^ Department of Pharmacology and Nutritional Sciences, University of Kentucky, Lexington, KY, United States; ^2^ Division of Research, Department of Surgery, University of Kentucky, Lexington, KY, United States; ^3^ Division of Laboratory Animal Resources, University of Kentucky, Lexington, KY, United States; ^4^ Dr. Bing Zhang Department of Statistics, University of Kentucky, Lexington, KY, United States; ^5^ Department of Microbiology, Immunology, and Molecular Genetics, University of Kentucky, Lexington, KY, United States

**Keywords:** aging, adipose tissue, gamma delta T cells, migration, proliferation, apoptosis

## Abstract

γδ T cells are resident in visceral adipose tissue (VAT) where they show an age-associated increase in numbers and contribute to local and systemic chronic inflammation. However, regulation of this population and mechanisms for the age-dependent accumulation are not known. In this study, we identified a progressive trend of γδ T cell accumulation in VAT over the lifespan in mice and explored physiological mechanisms contributing to accumulation. Using isochronic parabiotic pairs of wild-type (WT) and T cell receptor delta knockout (TCRδ KO) mice at young and old age, we confirmed that VAT γδ T cells are predominately a tissue-resident population which is sustained in aging. Migration of peripheral γδ T cells into VAT was observed at less than 10%, with a decreasing trend by aging, suggesting a minor contribution of recruitment to γδ T cell accumulation with aging. Since tissue-resident T cell numbers are tightly regulated by a balance between proliferation and programmed cell death, we further explored these processes. Using *in vivo* EdU incorporation and the proliferation marker Ki67, we found that the absolute number of proliferating γδ T cells in VAT is significantly higher in the aged compared to young and middle-aged mice, despite a decline in the proportion of proliferating to non-proliferating cells by age. Analysis of apoptosis via caspase 3/7 activation revealed that VAT γδ T cells show reduced apoptosis starting at middle age and continuing into old age. Further, induction of apoptosis using pharmacological inhibitors of Bcl2 family proteins revealed that VAT γδ T cells at middle age are uniquely protected from apoptosis via a mechanism independent of traditional anti-apoptotic Bcl2-family proteins. Collectively, these data indicate that protection from apoptosis at middle age increases survival of tissue-resident γδ T cells resulting in an increased number of proliferative cells from middle age onward, and leading to the age-associated accumulation of γδ T cells in VAT. These findings are important to better understand how adipose tissue dysfunction and related changes in the immune profile contribute to inflammaging among the elderly.

## 1 Introduction

γδ T cells are the prototype “unconventional” T cell, representing a unique subset that possesses characteristics of both innate and adaptive immunity (Vantourout and Hayday, 2013; Chien et al., 2014). They have been widely studied in the context of infections and autoimmune diseases (Bank, 2020; Kabelitz, 2020; Giri and Lal, 2021). However, their localization in peripheral tissues indicates physiological roles in maintaining tissue homeostasis beyond host protection (Johnson et al., 2020; Giri and Lal, 2021; Ribot et al., 2021). γδ T cells are uniquely enriched in adipose tissues (Kohlgruber et al., 2018) and have been identified as regulators of adipogenesis, thermogenesis, and sympathetic innervation (Zuniga et al., 2010; Kohlgruber et al., 2018; Hu et al., 2020). Research using diet-induced obesity models have provided evidence for a deleterious role of γδ T cells in driving obesity-induced inflammation and metabolic dysfunction (Mehta et al., 2015). However, under homeostatic conditions and after short-term ketogenic diet, γδ T cells in visceral adipose tissue (VAT) appear to be metabolically protective (Goldberg et al., 2020).

Adipose tissue has gained much interest in the aging field over the past decade as it appears to play a major role in age-related comorbidities (Huffman and Barzilai, 2009; Tchkonia et al., 2010; Palmer and Kirkland, 2016; Stout et al., 2017; Ou et al., 2022). Age-associated adipose tissue dysfunction results from a combination of adipose tissue redistribution, immune cell infiltration and functional alteration, decline in adipogenesis, preadipocyte senescence, reduced mi-RNA processing, and a decrease in brown and beige fat function (Palmer and Kirkland, 2016; Ou et al., 2022; Zhang et al., 2023). These changes collectively impart a profound impact on metabolic and cardiovascular health (Palmer and Kirkland, 2016; Stout et al., 2017; Reyes-Farias et al., 2021; Von Bank et al., 2021; Zhang et al., 2023). With respect to immune cell alterations, studies have linked age-associated VAT inflammation and dysfunction to disruptions in regulatory T cell, B cell, ILC2, eosinophil, and macrophage populations (Lumeng et al., 2011; Garg et al., 2014; Bapat et al., 2015; Camell et al., 2017; Trim et al., 2018; Camell et al., 2019; Brigger et al., 2020; Goldberg et al., 2021; Dahlquist and Camell, 2022). Nevertheless, the roles of immune cells in age-related adipose tissue dysfunction are complex and incompletely understood.

We recently reported that γδ T cells are increased by aging in VAT of mice and humans (Bruno et al., 2022). This increase occurs independent of adiposity and without regard for sex. In the absence of γδ T cells (TCRδ KO mice), inflammation, both locally in VAT and systemically, is reduced in aged mice confirming a role for these cells in age-associated inflammation (Bruno et al., 2022). Deficiency of γδ T cells in old age also improved the metabolic phenotype characterized by increased respiratory exchange ratio. However, the underlying mechanisms contributing to an age-associated accumulation of γδ T cells in VAT remain unclear.

The objective of this study was to investigate the contributions of different physiological mechanisms to age-associated γδ T cell accumulation in VAT. We first evaluated the trend of accumulation of γδ T cells over the lifespan in mice and explored whether increased recruitment of peripheral γδ T cells to VAT contributes to age-associated accumulation. In the absence of this finding, we shifted our focus to the tissue-resident population. T cell numbers are tightly regulated within tissues by a balance between proliferation and programmed cell death (Ginaldi et al., 2000); thus, we evaluated these two driving forces as mechanisms to increase the number of γδ T cells in VAT with aging.

Overall, our findings shed light on specific age-related immune alterations regarding γδ T cells in VAT. Since shifts in the resident immune profile contribute to adipose tissue dysfunction, inflammaging, and downstream metabolic perturbations, which increase the risk of chronic disease, these results aid in our understanding of the roles γδ T cells play in these processes.

## 2 Methods

### 2.1 Animals and husbandry

Male and female C57BL/6 mice were obtained from The Jackson Laboratory (Stock 664) or the National Institute on Aging. T cell receptor delta chain knockout mice (TCRδ KO, B6.129P2-Tcrdtm1Mom/J, Stock 2120) were obtained from The Jackson Laboratory and bred in-house. Different age groups of mice were utilized ranging from 2 to 24 months; age is specified for each experiment in figures and/or legends. Mice were housed in pressurized intraventilated cages and maintained in an environment under controlled temperature (21°C–23°C), humidity (30%–70%), and lighting (14 h/10 h, light/dark) with free access to drinking water and chow (Teklad Global No. 2918). All procedures were approved by the Institutional Animal Care and Use Committee at the University of Kentucky and performed in accord with the National Institutes of Health guidelines for ethical animal treatment.

### 2.2 Parabiosis

Isochronic parabiotic pairs were constructed using age- and sex-matched WT and TCRδ KO male and female mice (male to male or female to female). Young males were cohoused at weaning. Aged males were acclimated for pairing using an in-house developed clear plexiglass barrier for 2 weeks (Supplementary Figure S1). After removal of the barrier, pairs were cohoused for an additional 2 weeks before surgery to assure compatibility. Female mice were cohoused for at least 2 weeks prior to surgery without the barrier. Surgeries were performed following the protocol described by Kamran et al. (2013). Briefly, C57BL/6 and TCRδ KO mice were anesthetized with isoflurane, 4%–5%, provided by a precision vaporizer followed by application of ophthalmic ointment, meloxicam (5 mg/kg s. c), buprenorphine SR-LAB (Zoopharm, 1 mg/kg s.c.), and enrofloxacin (10 mg/kg, s.c.). The appropriate side of each mouse was clipped of fur and the mice transferred to a heated surgical platform with two separate side-by-side nose cones, with isoflurane maintained at 1.5%–2%. With the mice positioned in lateral recumbency, back-to-back, the exposed side of each mouse was aseptically prepped using betadine and isopropyl alcohol, and an incision was made through the skin extending from 5 mm above the elbow to 5 mm below the knee joint. The skin adjoining the incision was gently separated from underlying tissue to create a 5 mm flap surrounding the incision and the exposed joints were joined using 3-0 Prolene suture. The skin along the ventral and dorsal sides of the incision was then approximated and joined with 5–0 Vicryl suture in a continuous pattern. Subsequently, the mice were transferred to a recovery cage, administered 1 mL warm sterile physiological saline (s.c.), and monitored until recovered from anesthesia and were ambulatory. Additional doses of meloxicam were provided at 24- and 48-h post-op and remaining exposed sutures were removed at 14 days. Parabiotic pairs were euthanized at 4 weeks to harvest blood and tissues for flow cytometry analysis.

### 2.3 Euthanasia and sample collection

Mice were deeply anesthetized by isoflurane inhalation (5%), laparotomy performed, and blood collected from the inferior vena cava (IVC) by syringe needle with 10% volume of 0.1 M sodium citrate. Subsequently, the IVC was cut, and the entire vasculature was perfused with 30 mL physiological saline through the cardiac ventricles to eliminate circulating cells. For parabiotic pairs, perfusion was initiated from the heart of one mouse and the IVC of the other mouse was cut to visualize combined blood flow. For flow cytometry, fresh tissues including visceral adipose tissue (VAT) from the gonadal fat pads, spleen, liver (a portion of left lateral lobe) and lymph nodes (inguinal and brachial) were dissected and kept on ice until processing.

### 2.4 Tissue processing for single-cell suspensions

VAT, spleen, and blood were processed as previously described (Bruno et al., 2022). Liver and lymph nodes were pressed through a 100-μm cell strainer with a 5 mL syringe plunger, washed with 20 mL digestion buffer (0.5% BSA in HBSS with Ca^2+^Mg^2+^) and centrifuged at 500 × g for 10 min. RBC lysis was performed for liver (BioLegend 420302), followed by passing through a 70-μm strainer. After a second round of centrifugation, cells were resuspended in digestion buffer for subsequent counting, and processing for flow cytometry.

### 2.5 Flow cytometry

Single-cell suspensions in 250 µL of Dulbecco’s Phosphate Buffered Saline (DPBS, Gibco 14190-144) were stained with Fixable Viability Dye eFluor 450 (eBioscience 65-0843-14) according to the manufacturer’s protocol, and Fc receptor blocking was performed using TruStain FcX (Biolegend 156604) for 10 min on ice in 250 µL of DPBS containing 1 mM EDTA, 25 mM HEPES, 1% FBS. Cells were further incubated for 30 min at 4°C in the dark with respective antibodies for cell surface staining (Supplementary Table S1). For cell surface analyses only, stained cells were fixed with 4% paraformaldehyde (Biolegend 420801) for 20 min. For intracellular staining, SVF cells were permeabilized following surface staining according to standard protocol (Invitrogen 00-5523-00), and intracellular staining was performed using antibodies for Ki-67 and isotype control. Cells were analyzed on a FACSymphony A3 Cell Analyzer (BD, San Jose, CA, United States). Analysis of flow cytometry data was performed using the FlowJo data analysis software (FlowJo, LLC, Ashland, OR, United States).

### 2.6 *In vivo* cell proliferation study with EdU

Stock solution of 5-ethynyl-2′-deoxyuridine (EdU) at 40 mg/mL was prepared by dissolving 10 mg in 250 µL of DMSO which was stored at −20°C. Each mouse was injected with 1 mg (Chen et al., 2019) dosage using 25 µL of stock solution and 375 µL PBS to make 400 µL of total injection volume. Mice were given intraperitoneal injections once daily, 24 h apart, for 3 consecutive days and sacrificed on day four. Body weights were measured before and after 3 injections and did not change significantly. VAT was harvested and processed to obtain single-cell suspensions which were stained with Fixable Viability Dye eFluor 450 and antibodies against cell surface markers (Supplementary Table S1), followed by EdU staining according to manufacturer’s guidelines using the Click-iT Plus EdU Alexa Fluor 594 flow cytometry assay kit (Invitrogen C10646).

### 2.7 Analysis of apoptosis

VAT and lymph nodes were collected from mice and processed to obtain single-cell suspensions. Cells were resuspended in DPBS containing 1 mM EDTA, 25 mM HEPES, 1% FBS, and Fc receptor blocking was performed followed by surface staining. Cells were then stained with CellEvent™ Caspase-3/7 Green Flow Cytometry Assay kit according to manufacturer’s protocol (Invitrogen C10740). In order to induce apoptosis, cells (∼1 × 10^6^) were incubated with 2 µM ABT737 (Selleck chem S1002) and 1.6 µM Mcl-1 Inhibitor II (Sigma-Aldrich 5.08053) or 0.2% DMSO (vehicle) for 3 h at 37°C with 5% CO_2_ similar to the protocol described by Tan et al. (2019). Subsequently, cells were washed with DPBS containing 1 mM EDTA, 25 mM HEPES, 1% FBS and Fc receptor blocking, cell surface staining, and staining for caspase 3/7 performed as described above.

### 2.8 Statistical analysis

Continuous bivariate associations were analyzed using simple linear regression. Categorical explanatory variables with a continuous response were analyzed using one-way ANOVA with or without repeated measures as appropriate. Post-hoc pairwise comparisons were done using Tukey’s Honest Significant Difference for independent samples and paired t-tests for repeated measures. Normality with constant variance was assumed in all analyses. No evidence of violations of normality were detected.

## 3 Results

### 3.1 γδ T cells progressively accumulate in visceral adipose tissue over the lifespan in mice

**FIGURE 1:**
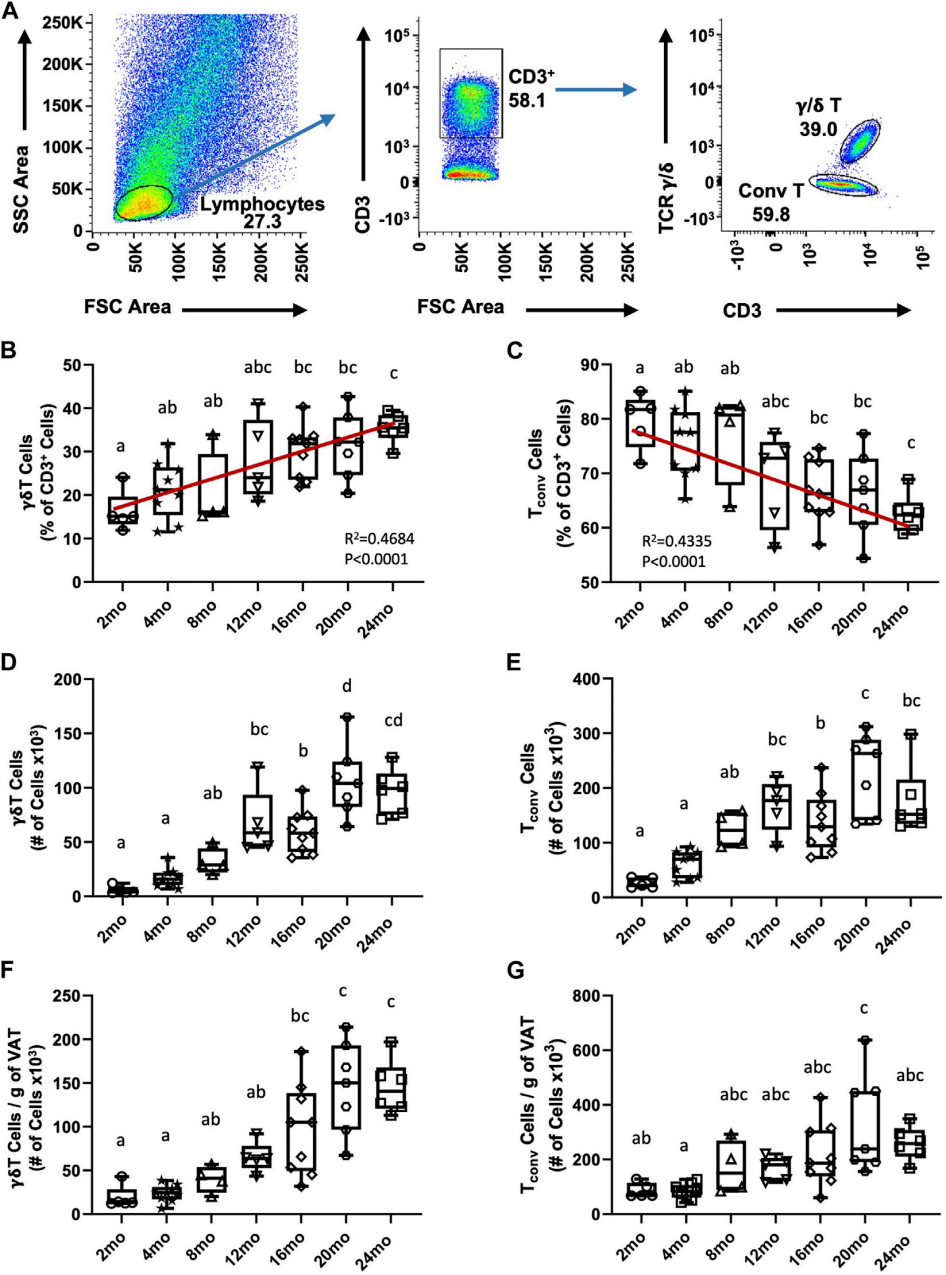
Accumulation of γδ T cells and Tconv cells in visceral adipose tissue over the lifespan in mice. Epididymal adipose tissues were harvested from mice and single-cell suspensions of SVF cells prepared for FACS analyses. **(A)** Representative flow cytometry plots of γδ T and T_conv_ cells among CD3^+^ lymphocytes in VAT. **(B,C)** Percentage and **(D,E)** Total number of γδ T and T_conv_ cells were quantified among the total CD3^+^ lymphocyte population in 2 months (*n* = 5), 4 months (*n* = 9), 8 months (*n* = 4), 12 months (*n* = 5), 16 months (*n* = 9), 20 months (*n* = 7), and 24 months (*n* = 6) old male C57BL/6 mice. **(F,G)** Number of cells adjusted per gram of VAT. Data are expressed in box plots from minimum to maximum values with bars representing the mean; each symbol represents an individual mouse. Statistical differences were determined by one-way ANOVA with Tukey’s Honest Significant Difference for multiple comparisons. Age groups not connected by the same letters (a–d) are significantly different. The correlation over the lifespan was determined using Pearson’s Correlation Coefficient. g: gram; mo: month; T_conv_: conventional T cell; VAT: visceral adipose tissue.

We previously reported an age-associated increase in γδ T cells in aged (19–24 months) compared to young (4–5 months) mice (Bruno et al., 2022); however, at what age this becomes apparent was not determined. To identify the trend of γδ T cell accumulation in VAT over the lifespan, γδ T cells were evaluated in seven age groups of mice (2 months, 4 months, 8 months, 12 months, 16 months, 20 months, 24 months). CD3^+^ T cells were quantified among lymphocytes and γδ T cells were distinguished by their TCR expression; conventional T cells (T_conv_, i.e., αβ T cells) were evaluated in parallel (Figure 1A). The entire flow cytometry gating scheme is shown in Supplementary Figures S2A. Among CD3^+^ lymphocytes, the percentage of γδ T cells progressively increased in VAT with aging (Figure 1B, *R* ^2^ = 0.4684, *p* < 0.0001), while the percentage of T_conv_ cells reciprocally declined with age (Figure 1C, *R* ^2^ = 0.4335, *p* < 0.0001). The total number of γδ T cells showed a significant increase at middle age (12–16 months) which further increased at old age (20–24 months) (Figure 1D). The number of T_conv_ cells likewise significantly increased at middle age (12–16 months); however, no further significant increase at old age was observed (Figure 1E). To understand if adiposity plays a role in the age-related accumulation of T cells in VAT, the number of cells was adjusted for fat mass (see Supplementary Figures S3A, B for body weight and fat mass data). In the resulting data, a significant increase in γδ T cells at old age remained (Figure 1F), indicating that age-related γδ T cell accumulation is adiposity-independent. In contrast, statistical significance for the increase was lost for T_conv_ cells (Figure 1G), which points to adiposity as a major determinant for T_conv_ accumulation in VAT over the lifespan. Furthermore, the significant increase in γδ T cells at middle age was also lost after fat mass adjustment, indicating a contribution of mid-age weight gain to γδ T cell abundance. In comparison, aging minimally influenced abundance of γδ T cells and T_conv_ cells in peripheral lymph nodes (Supplementary Figures S4A–D). Collectively, these data indicate that γδ T cells progressively accumulate in VAT over the lifespan and that the late age-associated increase is independent of adiposity and unique to this tissue-resident population of cells.

### 3.2 Recruitment of γδ T cells to VAT is not enhanced by aging

**FIGURE 2:**
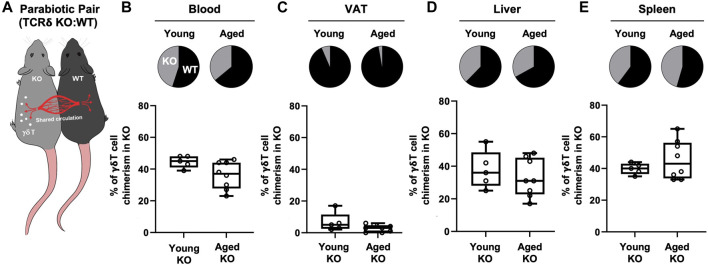
Recruitment of γδ T cells to VAT is not enhanced by aging. γδ T cell chimerism was evaluated 4 weeks after parabiosis surgery in young (*n* = 5 WT:TCRδ KO pairs, 5–9 months old males) and aged (*n* = 4 male pairs and *n* = 4 female pairs, 21–24 months old) parabiotic pairs. Data from males and females were combined as no statistical differences between the sexes were found. **(A)** Representative diagram of WT:TCRδ KO isochronic parabiotic pairs. Average γδ T cell chimerism in **(B)** Blood **(C)** VAT, **(D)** Liver, and **(E)** Spleen for each genotype (WT and TCRδ KO) of the parabiotic pair was assessed by flow cytometry. Pie charts indicate the average proportion of γδ T cells in each genotype of the parabiotic pair for both age groups. Proportions were calculated as the percentage of γδ T cells in the KO (or WT) mouse over the sum of γδ T cells in total among the pair. Box plots show percent of γδ T cells present in the TCRδ KO mouse of the pair at each age group. Each symbol represents an individual mouse, with bars representing the mean. Statistical differences were determined by two sample t-tests, no significant differences were observed. VAT: visceral adipose tissue; WT: wild type; TCRδ KO: T cell receptor delta knock out.

Previous studies using parabiosis of CD45.1 and CD45.2 congenic mice have reported VAT γδ T cells in young mice to be a tissue-resident population (Kohlgruber et al., 2018; Goldberg et al., 2020). However, since adipose tissue is chronically inflamed in aging, promoting the secretion of inflammatory cytokines and chemokines, we tested the hypothesis that the inflamed microenvironment of aged VAT provides recruitment signals for peripheral γδ T cells. Isochronic parabiotic pairs of wild-type (WT) and TCRδ KO mice at both young and old age were used to evaluate peripheral γδ T cell migration into VAT (Figure 2A). γδ T cells in blood showed comparable chimerism between WT and TCRδ KO mice at both ages (Figure 2B, young: 55.4% ± 3.8% in WT vs. 44.6% ± 3.8% in KO; aged: 64.1% ± 8.6% in WT vs. 36% ± 8.6% in KO), confirming distribution of circulating cells and validating our model system. In the young pairs, minimal recirculation of γδ T cells was observed into the VAT of the TCRδ KO mouse from its WT pair (Figure 2C, 6.6% ± 6% in young TCRδ KO), confirming previous reports that VAT γδ T cells are tissue-resident (Kohlgruber et al., 2018; Goldberg et al., 2020). Similar data were observed in the aged pairs (2.75% ± 2.3% γδ T cells in the aged TCRδ KO mouse, *p* = 0.1239 vs. young KO), indicating that the aged microenvironment does not enhance γδ T cell accumulation. This is likely to be unique to VAT as we observed recirculation in liver (Figure 2D) and spleen (Figure 2E). Therefore, while there is continuous low-level migration of γδ T cells into VAT (<10%), which could contribute to accumulation over the lifespan, recruitment of these cells is not enhanced by aging.

### 3.3 Proliferation of VAT-resident γδ T cells decreases with aging although the total number of proliferating cells is expanded in the aged

**FIGURE 3:**
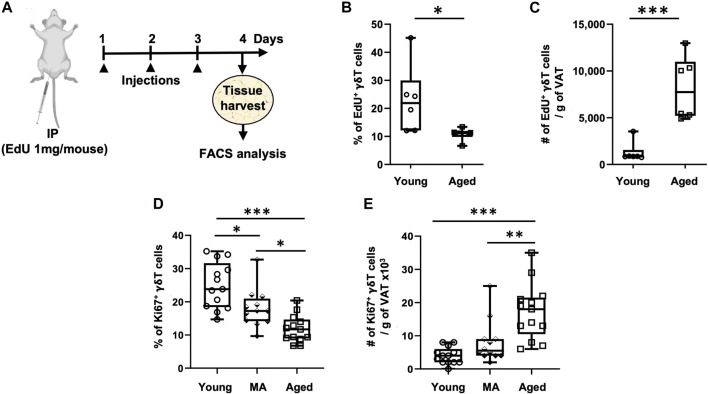
Proliferation of VAT-resident γδ T cells decreases with aging. Proliferation was assessed *in vivo* and *ex vivo* by EdU incorporation and Ki67 positivity, respectively. **(A)** Experimental design for EdU incorporation in young (*n* = 6, 4 months), and aged (*n* = 6, 23 months) male mice. Epididymal adipose tissues were harvested and single-cell suspensions prepared after three consecutive days of EdU injections. **(B)** Percentage and **(C)** Number of EdU^+^ γδ T cells per Gram of VAT were quantified using Click-iT Plus EdU Flow Cytometry Assay kit. In separate mice, epididymal adipose tissues were harvested for intracellular Ki67 staining by flow cytometry. **(D)** Percentage and **(E)** Number of Ki67^+^ γδ T cells per Gram of VAT in young (*n* = 13, 4–6 months), middle aged (MA, *n* = 12, 12–16 months) and aged (*n* = 13, 21–25 months) male mice. Data are expressed in box plots from minimum to maximum values with bars representing the mean; each symbol represents an individual mouse. Statistical differences were determined by one-way ANOVA with Tukey’s Honest Significant Difference for multiple comparisons. *p < 0.05, **p < 0.01, ***p < 0.001. IP: Intraperitoneal injection; EdU: 5-ethynyl-2′-deoxyuridine; VAT: visceral adipose tissue.

Since *de novo* generation of γδ T cells exists only in the thymus and peripheral recruitment to the VAT is minimal and not affected by aging, we evaluated whether proliferation of tissue-resident γδ T cells is enhanced in the aged. Two different approaches were utilized: *in vivo* EdU incorporation (Figure 3A) and intracellular staining for the proliferation marker Ki67 (flow cytometry gating schemes are shown in Supplementary Figure S2). The percentage of EdU^+^ γδ T cells significantly declined from young to aged (Figure 3B, 23% ± 12% in young vs. 11% ± 2% in aged, *p* = 0.0367), indicating that the proportion of proliferating to non-proliferating γδ T cells decreases by aging. The total number of EdU^+^ γδ T cells per gram of VAT, however, showed a significant 6.3-fold increase in the aged (Figure 3C, *p* = 0.0008). Note that mass of VAT was not significantly different between young and aged mice in this study (data not shown). T_conv_ cells in VAT showed similar results (Supplementary Figures S5A, B).

We next sought to validate these results utilizing the proliferation marker Ki67, in doing so we added a middle-aged group. The percentage of Ki67^+^ γδ T cells progressively declined from young to old age (Figure 3D, 25% on average in young, 18% in middle age, and 12% in old age, *p* < 0.001 for young vs. aged comparison). The total number of Ki67^+^ γδ T cells per gram of VAT, however, showed a significant 4.4-fold increase in the aged group only (Figure 3E, *p* < 0.0001: young vs. aged, *p* = 0.0025: middle-aged vs. aged). In T_conv_ cells, the percentage of Ki67^+^ cells declined from young to middle age without further change at old age; however, the total number of Ki67^+^ T_conv_ cells per gram of VAT showed a significant 5.6-fold increase in only the aged group without any significant difference between young and middle-aged (Supplementary Figures S5C, D). In peripheral lymph nodes, the percentage of Ki67^+^ γδ T cells remained unchanged over the lifespan, while there was a slight increase in total number in the aged (Supplementary Figures S6A, C). T_conv_ cells in peripheral lymph nodes, on the other hand, showed a slightly higher proportion of Ki67^+^ cells in the aged but no change in total number (Supplementary Figures S6B, D).

Collectively, these results indicate that although the proportion of proliferating to non-proliferating γδ T cells decreases with aging, the total number of proliferating cells within the population increases in old age likely on account of the population itself being expanded over the lifespan. Further, both γδ T cells and T_conv_ cells in VAT show similar results with respect to the effect of age on proliferation, while cells in the peripheral lymph nodes were minimally affected.

### 3.4 γδ T cells are protected from apoptosis beginning at the middle age

**FIGURE 4:**
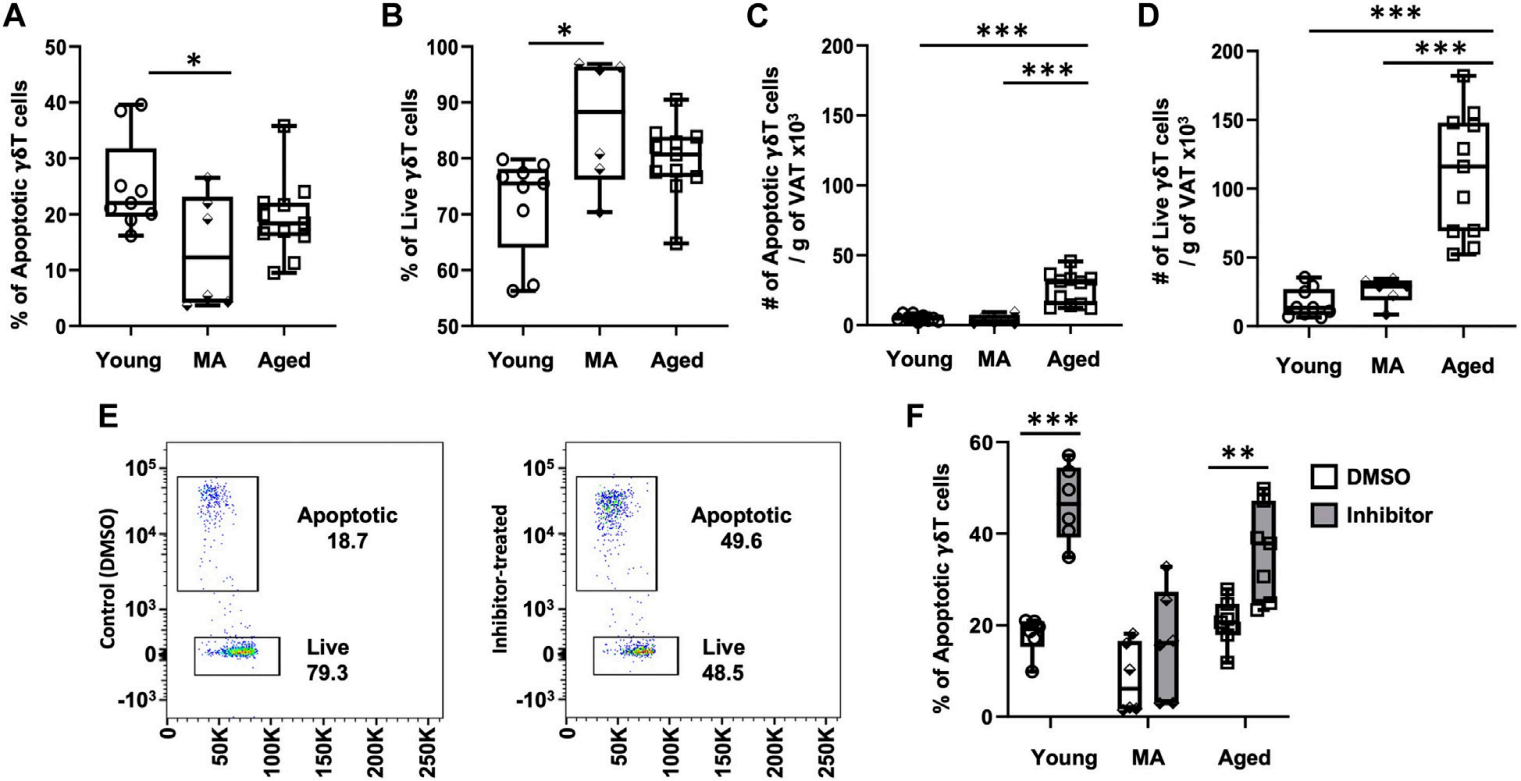
γδ T cells in VAT are protected from apoptosis beginning at middle age. Epididymal adipose tissues were harvested from mice and single-cell suspensions of SVF cells prepared for FACS analyses using CellEvent Caspase 3/7 Flow Cytometry Assay with cell surface staining to identify γδ T cells. **(A,B)** Percentage and **(C,D)** Number per gram of VAT of apoptotic and live γδ T cells in young (*n* = 9, 4 months), middle age (MA, *n* = 6, 12 months) and aged (*n* = 11, 22 months) male mice. Data are expressed in box plots from minimum to maximum values with bars representing the mean; each symbol represents an individual mouse. Statistical differences were determined by one-way ANOVA with Tukey’s Honest Significant Difference for multiple comparisons. In cells from separate mice, apoptosis was induced by incubation with Bcl2-family inhibitors ABT737 and Mcl-1 inhibitor II for 3 h. **(E)** Representative flow cytometry plots of γδ T cells treated with DMSO (control) and Bcl2 inhibitors. **(F)** Apoptotic γδ T cells in young (*n* = 6, 4 months), middle age (MA, *n* = 6, 12 months) and aged (*n* = 7, 22 months) male mice. Data are expressed in box plots from minimum to maximum values with bars representing the mean; each symbol represents an individual sample. Pairwise statistical differences were detected by paired *t*-test within each age group, *p < 0.05, **p < 0.01, ***p < 0.001. g: gram; MA: middle-aged; VAT: visceral adipose tissue.

To understand whether aging influences VAT-resident γδ T cell turnover, we analyzed apoptosis via caspase 3/7 activity (FACS gating scheme shown in Supplementary Figures S7). The proportion of apoptotic γδ T cells significantly declined at middle age compared to young without any further change in the aged (Figure 4A, 25% ± 8% in young, 13 %± 10% in middle-aged, 19% ± 7% in aged, *p* = 0.0363 young vs. middle-aged, *p* = 0.3685 middle-aged vs. aged). A concomitant increase in the proportion of live cells was observed starting at middle age (Figure 4B, *p* = 0.0117 young vs. middle-aged, *p* = 0.2993 middle-aged vs. aged), suggesting that γδ T cells are protected from apoptosis from middle age onwards. However, the absolute number of apoptotic and live γδ T cells per gram of VAT was significantly higher in the aged (Figures 4C, D, *p* < 0.001 young vs. aged and *p* < 0.001 middle-aged vs. aged).

To address the role of anti-apoptotic proteins in VAT-resident γδ T cell survival, apoptosis was induced by treating SVF cells isolated from adipose tissues with a combination of Bcl-2 family inhibitors: ABT737 (inhibitor of Bcl-2, Bcl-xl, and Bcl-w) and Mcl-1 inhibitor II (specific to Mcl-1). Apoptosis via caspase 3/7 activity in γδ T cells was compared between control (treated with DMSO) and inhibitor-treated cells (Figure 4E) by FACS with cell surface staining for γδ T cells. In the presence of inhibitors, apoptosis was significantly induced in young γδ T cells (Figure 4F, 18% ± 4% to 45% ± 8%, *p* = 0.0008). In contrast, apoptosis was not significantly induced in cells from middle-aged mice (8% ± 8% to 16% ± 12%, *p* = 0.1062). γδ T cells from aged mice were responsive to inhibitor treatment with apoptosis induced from 20% ± 5% to 36% ± 10% (*p* = 0.0011), although induction of apoptosis appeared mildly blunted compared to young. These data suggest that γδ T cells at middle age may be protected from apoptosis via a mechanism unrelated to the targeted Bcl2 family of anti-apoptotic proteins.

VAT-resident T_conv_ cells likewise showed a significant decline in the proportion of apoptotic cells from young to middle age, suggesting protection from apoptosis similar to γδ T cells (Supplementary Figures S8A, B). The absolute number of apoptotic and live T_conv_ cells after adjusting for fat mass also showed similar results to γδ T cells with numbers increasing only in the old age group (Supplementary Figures S8C, D). However, in the presence of Bcl2 family inhibitors, induction of apoptosis was significant in all groups (Supplementary Figures S6E), indicating a unique pro-survival mechanism for γδ T cells.

Contrarily, apoptosis was not reduced in γδ T cells or T_conv_ cells from the peripheral lymph nodes at middle age or old age. Instead a significant increase in the percent of apoptotic γδ T cells was observed from middle age to old age (Supplementary Figures S9A, B and Supplementary Figures 10A, B). The total number of apoptotic or live γδ T cells in lymph nodes did not significantly change over the lifespan (Supplementary Figures S9C, D); however, T_conv_ cells showed significant changes by aging (Supplementary Figures S10C, D). Apoptosis of lymph node γδ T cells and T_conv_ cells was enhanced in all age groups in the presence of Bcl2-family inhibitors although the level of apoptotic cells was already high at baseline in these cells (Supplementary Figures S9E, 10E).

Collectively, our data indicate that T cells (both γδ T and Tconv) are uniquely protected from apoptosis in VAT, beginning at middle age. Further, the presence of a distinctive mechanism for VAT-resident γδ T cells exists which is independent of the traditional Bcl2-family of anti-apoptotic proteins.

## 4 Discussion

**FIGURE 5:**
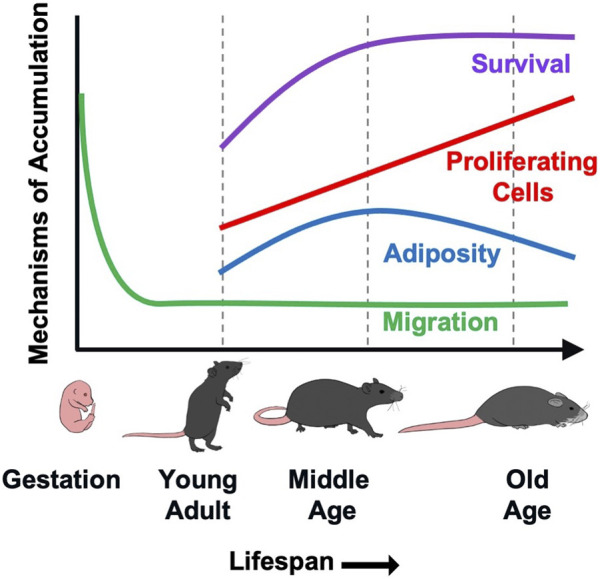
A putative model demonstrating the contribution of various mechanisms to γδ T cells accumulation in VAT with aging. γδ T cells are generated in the fetal thymus followed by waves of egression to peripheral sites. Migration of γδ T cells into VAT occurs at minimal levels (<10%) over the lifespan, but is not increased by aging. Rather, protection from apoptosis beginning at middle age leads to increased survival of these long-lived cells. This, in combination with a low level of recruitment over the lifespan leads to the expanded population which continues to proliferate via clonal expansion into old age. The increase in proliferative cell numbers occurs despite an overall reduction in proliferation across the lifespan. Adiposity also appears to play a role during the period of middle age weight gain, but is no longer a factor at old age.

Our prior work demonstrated that accumulation of γδ T cells in VAT contributes to age-associated chronic inflammation by increasing IL-6 production from resident non-immune stromal cells (Bruno et al., 2022). In the current study, we aimed to understand the underlying cause of γδ T cell accumulation in VAT with aging. Our data suggest that multiple processes are likely at play which cumulatively lead to γδ T cell expansion over the lifespan (Figure 5). Using a parabiosis model, we demonstrated that recruitment of γδ T cells to VAT is not increased by aging. Rather, protection from apoptosis beginning at middle age, in combination with a low level of migration into VAT over the life span, leads to the expanded population which continues to proliferate via clonal expansion into old age. Adiposity also potentially plays a role during the period of middle age weight gain, but is no longer a factor at old age. These findings highlight the complexity of age-related immune changes which impact homeostasis during the aging process and contribute to the development of chronic disease and dysfunction in old age.

While our previous work showed that VAT-resident γδ T cells are increased by aging in mice, those data were limited to two age groups: 4–5 months-old and 19–24 months-old (Bruno et al., 2022). In the present study, we used seven different age groups of mice to understand whether the expansion was linear over the lifespan or a late-age event. Our data revealed a progressive increase of γδ T cells in VAT over the lifespan. The total number of γδ T cells showed an initial increase in VAT at middle age (12–16 months) with continued increase into old age (20–24 months). However, after adjusting for fat mass, the significance of the increase was lost in the middle-aged mice, but maintained in the older mice. This suggests that adiposity plays a role in γδ T cell expansion into middle age, but the continued expansion from mid- to old age is adiposity-independent. This is substantiated by a plateau in the weight of the fat pads (epididymal) and body weight from 12 months onwards. Short-term high-fat diet has been shown to increase the number of γδ T cells in proportion to the increase in fat mass (Mehta et al., 2015; Bruno et al., 2022); thus, middle age weight gain could account for this finding. The increase in number of T_conv_ cells, on the other hand, lost significance for all groups after fat mass adjustment, except 20 months, suggesting more reliance on adiposity for expansion. Consequently, γδ T cells show a unique pattern of expansion in old age. As our previous work identified a deleterious role for γδ T cells with regard to chronic age-associated inflammation and metabolic dysfunction (Bruno et al., 2022), understanding the mechanism of expansion provides important information.

Although recent studies have found VAT γδ T cells to be a tissue-resident population in young mice (Kohlgruber et al., 2018; Goldberg et al., 2020), we initially hypothesized that γδ T cells would be recruited to VAT during aging due to the chronically inflamed microenvironment. This seemed logical as pro-inflammatory cytokines and chemokines have been shown to recruit γδ T cells into several other tissues (Xu et al., 2021). Moreover, the number of circulating γδ T cells declines in aged mice (Bruno et al., 2022) and humans (Romano et al., 2000; Argentati et al., 2002; Colonna-Romano et al., 2002; Clark and Thomas, 2020; Singh et al., 2022), suggesting the potential for redistribution to VAT. However, this hypothesis proved to be incorrect. We assessed migration of γδ T cells in isochronic parabiotic pairs of WT and TCRδ KO mice at both young and old age by identifying the number of γδ T cells which were able to recirculate and take up residence in the tissues of the KO animal. While blood, liver, and spleen showed balanced levels of chimerism between the WT and TCRδ KO of the young pairs, minimal chimerism was observed of γδ T cells migrating into the VAT of the TCRδ KO from its WT pair. This did not change as a function of age, suggesting that the majority of γδ T cells remain a self-sufficient tissue-resident population over the lifespan. However, it is possible that this low level of migration contributes to γδ T cell accumulation in VAT over the lifespan. Our data in young mice are similar to that observed by others using young CD45.1:CD45.2 parabiotic pairs to assess γδ T cell migration to VAT (Kohlgruber et al., 2018; Goldberg et al., 2020). We recognize important limitations in this study such as our use of TCRδ KO mice which could lack the necessary recruitment signals for γδ T cells. However, this is unlikely since the cells were able to take up residence in other tissues. Further, it would be of interest to identify whether migration is enhanced during middle age when γδ T cell expansion shows some dependency on adiposity; however, we were unable to perform those studies due to our inability to maintain and age the KO strain at this time. It is possible that a migrating population contributes to accumulation during middle age weight gain.

Lack of age-dependent γδ T cell recruitment to VAT indicates that a tissue-specific mechanism must lead to γδ T cell expansion in old age. As *de novo* generation of γδ T cells exists only in the thymus (Sandrock et al., 2018; Parker and Ciofani, 2020; Ribot et al., 2021), we investigated two possible forces which maintain cellular homeostasis − proliferation and programmed cell death. The proportion of proliferating to non-proliferating γδ T cells progressively declined from young to old age, suggesting a decrease in the proliferative capacity by aging. This is not unexpected since proliferation is well-known to decrease in the aged (Makinodan and Adler, 1975; Lewis et al., 2022). However, the absolute number of proliferating γδ T cells within VAT showed a significant increase at old age compared to both young and middle-aged. This incongruity is simply due to the increased number of γδ T cells at old age - while among the total population, fewer γδ T cells are able to proliferate at old age compared to younger ages, there are more γδ T cells in total leading to higher numbers of both proliferating and non-proliferating cells. The increased number of γδ T cells at middle age likely serves as a reservoir for continued accumulation into old age via clonal expansion. However, proliferation was not increased at middle age prompting us to explore alterations in cell survival.

Our data show that γδ T cells in VAT are uniquely resistant to apoptosis at middle age. This trend continued into old age although statistical significance was lost, possibly due to larger variation which occurs naturally in older animals. A recent study showed that Vγ6^+^ γδ T cells in the skin are protected from apoptosis via the B-cell lymphoma 2-related protein A1 (Bcl2a1) family of proteins (Tan et al., 2019). Bcl2a1 proteins are a family including Bcl2a1a–Bcl2a1d which are not inhibited by treatment with ABT-737 or Mcl-1 inhibitors (Vogler, 2012). Using a similar experimental design as Tan *et al.*, we explored this possible mechanism for γδ T cell protection from apoptosis in VAT. Apoptosis was induced upon inhibition of Bcl-2, Bcl-xl, Bcl-w and/or Mcl-1 in young and aged VAT γδ T cells, suggesting a role for these Bcl2-family anti-apoptotic proteins in the maintenance of this cell population. It is important to note that the degree of apoptosis induction was more robust in the cells from young mice. In cells from aged mice, there was overlap between vehicle and inhibitor-treated cells suggesting variation in the degree of responsiveness among aged animals with some responding to inhibition and others not. However, apoptosis was not notable in VAT γδ T cells from middle-aged mice by traditional Bcl2-family inhibition indicating a unique mechanism potentially involving Bcl2a1 proteins in this group. The Bcl2a1a and Bcl2a1d genes were recently reported to be enriched in IL17A^+^ γδ T cells from CD18 (β_2_ integrin) KO mice, which display a striking increase in γδ T cell numbers in lung, uterus, spleen, and circulation (McIntyre et al., 2020). Although not measured in this study, an increase in anti-apoptotic Bcl2a1 proteins in γδ T cells, as occurs in γδ T cells from other tissues and model systems, could promote survival of VAT-resident γδ T cells at middle age and into old age. This finding was unique to VAT γδ T cells since T_conv_ cells in VAT, despite a similar decreasing trend in the proportion of apoptotic cells with age, responded to inhibitor treatment uniformly regardless of age. These similarities and differences among VAT-resident lymphocytes suggest the likelihood that cell-intrinsic properties in combination with age-related environmental changes contribute to differences in the regulation of apoptosis over the lifespan. In contrast, apoptosis of γδ T cells residing in peripheral lymph nodes was increased in old age indicating tissue-specific mechanisms which are likely environmental in nature.

A limitation of this work is the lack of specificity of our data for γδ T cell subtypes. Multiple subpopulations of γδ T cells exist due to V(D)J recombination of the 7 gamma and 6 delta chains (Legut et al., 2015). Mehta et al. detected the expression of Vγ1, Vγ2, Vγ4, Vγ6 and Vδ1, Vδ3, Vδ4 genes (Heilig & Tonegawa nomenclature (Heilig and Tonegawa, 1986)) in VAT of young mice by PCR (Mehta et al., 2015). Targeted flow cytometric analyses confirmed that VAT of young mice is primarily composed of Vγ6^+^ cells, which correspond to the more abundant CD3ε^hi^CD27^neg^PLZF^+^ IL-17A-producing population, while the CD3ε^lo^CD27^+^ PLZF^neg^ IFNγ-producing population appears to contain both Vγ1^+^ and Vγ4^+^ cells (Kohlgruber et al., 2018). It is possible that γδ T cell accumulation in VAT with aging is subpopulation specific. Indeed, Vγ6^+^ γδ T cells increased by aging in the peripheral lymph nodes of mice, while Vγ1 and Vγ4 populations showed an age-associated decrease (Chen et al., 2019). We were not able to identify different γδ T cell subpopulations by FACS as the commercially available antibodies, which we attempted to validate in our lab, lacked sufficient marker specificity (data not shown). LN and thymic Vγ6^+^ cells are believed to have higher motility and traffic between tissues under homeostatic conditions and in response to inflammation (Tan et al., 2019), which raises the possibility that certain subpopulations of γδ T cells migrate into VAT over the lifespan contributing in part to the expanded population. Protection from apoptosis as well as differences in proliferative capacity may also be subpopulation specific. Our future studies will delve into these details which will refine the generated knowledge.

In conclusion, γδ T cells showed a progressive trend of accumulation in VAT over the lifespan in mice which was adiposity-dependent at middle age but independent of adiposity at old age. In the absence of evidence that γδ T cells increasingly traffic to VAT with aging, we focused on mechanisms supporting tissue-resident cell homeostasis. Our findings indicate that γδ T cells in VAT are protected from apoptosis from middle age onwards contributing to enhanced cell survival. This, in combination with an increased number of proliferating cells in the aged due to clonal expansion, not only maintains the population, but leads to the observed age-associated accumulation of γδ T cells. We therefore propose that the processes leading to γδ T cell accumulation are set into motion at middle age and not rescued into old age due to the multiple mechanisms at play. These findings are important to better understand how immune cells mediate adipose tissue dysfunction with aging and may aid in the identification of future novel therapeutic interventions to reduce the burden of inflammaging-associated diseases among the elderly.

## Data Availability

The raw data supporting the conclusions of this article will be made available by the authors, without undue reservation.
