# Supplementary material for: Mechanisms of γδ T cell accumulation in visceral adipose tissue with aging
Source: Front Aging. 2024 Jan 11;4:1258836. doi: 10.3389/fragi.2023.1258836 (PMC10808514; doi:10.3389/fragi.2023.1258836)
Supplement: Supplementary file 1 [file Table1.DOCX]

Supplementary Table 1. Antibodies used for the flow cytometry studies.

| **Antigen** | **Conjugate** | **Manufacturer** | **Identifier** |
| --- | --- | --- | --- |
| CD45 | APC/Cyanine7  Brilliant Violet 510 | BioLegend  BioLegend | 103116  103138 |
| CD3 | FITC  PE/Cyanine7 | BioLegend  BioLegend | 100204  100219 |
| TCRγ/δ | APC-Vio 770  PerCP-Vio 700  PerCP/Cy5.5 | Miltenyi Biotec Miltenyi Biotec  BioLegend | 130-126-042  130-117-665  118118 |
| Ki-67 | FITC | BioLegend | 652410 |
| IgG_2a_, κ  (Isotype for Ki-67) | FITC | BD Pharmingen | 20624A |
