# Supplementary material for: Mechanisms of γδ T cell accumulation in visceral adipose tissue with aging
Source: Front Aging. 2024 Jan 11;4:1258836. doi: 10.3389/fragi.2023.1258836 (PMC10808514; doi:10.3389/fragi.2023.1258836)
Supplement: Supplementary file 2 [file DataSheet1.PDF]

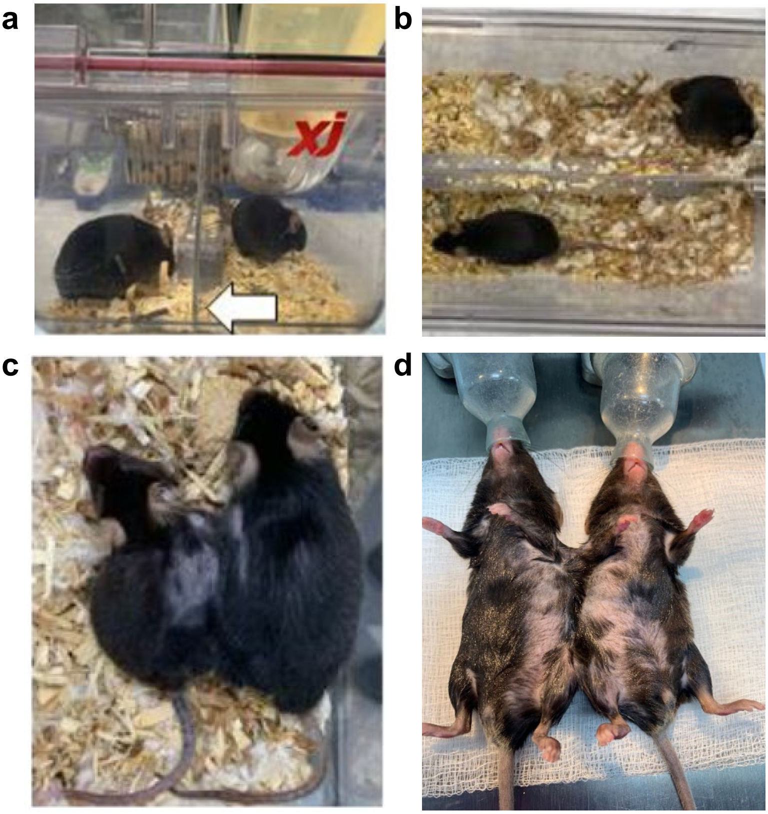


**Supplementary Figure 1. Acclimation of parabiotic pairs with barrier.** A clear plexi-glass barrier was constructed in-house to allow for acclimation of male mice to the same cage **(a)** front view **(b)** top view. Picture of **(c)** active parabiotic pairs after surgery and **(d)** at euthanasia.

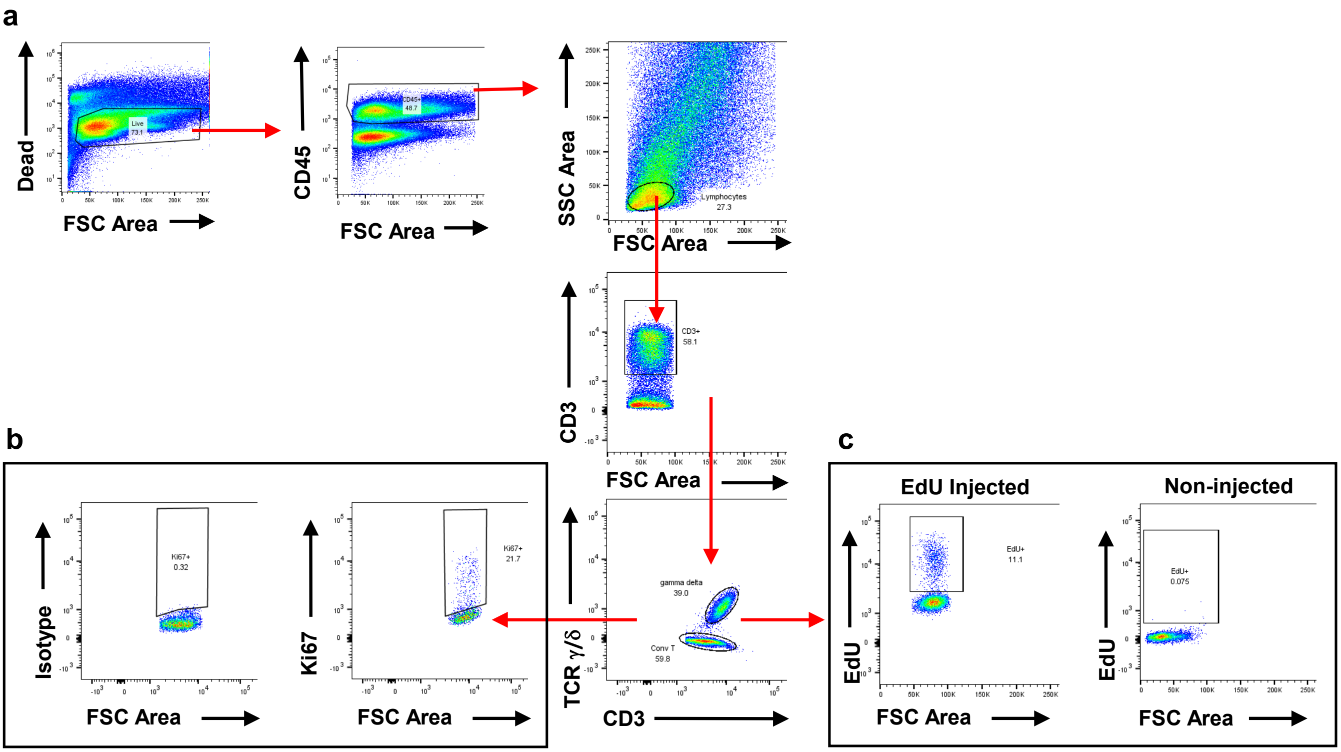


**Supplementary Figure 2.** **(a)** Representative flow cytometry plots to identify γδ T and $T_{conv}$ cells. **(b)** Gating scheme for Ki67+ γδ T and $T_{conv}$ cells. **(c)** Gating Scheme for EdU+ γδ T and $T_{conv}$ cells in EdU injected and non-injected mice.

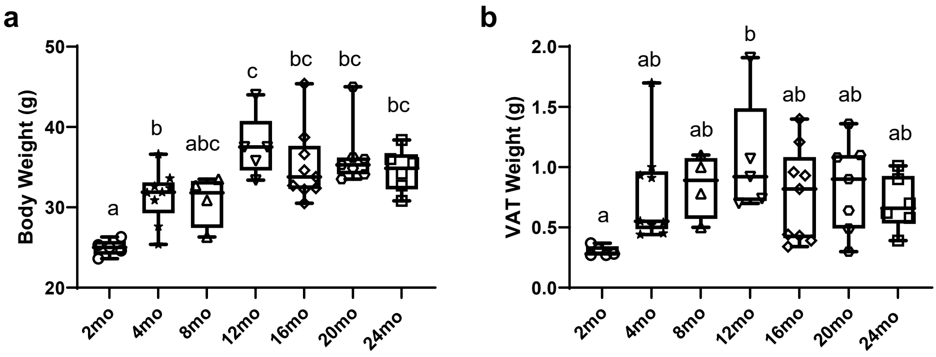


**Supplementary Figure 3. (a)** Body weight and **(b)** VAT weight in 2 mo (n=5), 4 mo (n=9), 8 mo (n=4), 12 mo (n=5), 16 mo (n=9), 20 mo (n=7), and 24 mo (n=6) old male C57BL/6 mice. Data are expressed in box plots from minimum to maximum values with bars representing the mean; each symbol represents an individual mouse. Statistical differences were determined by one way ANOVA with Tukey's Honest Significant Difference for multiple comparisons. Age groups not connected by the same letters (a,b,c) are significantly different. g: gram; mo: month; VAT: visceral adipose tissue.

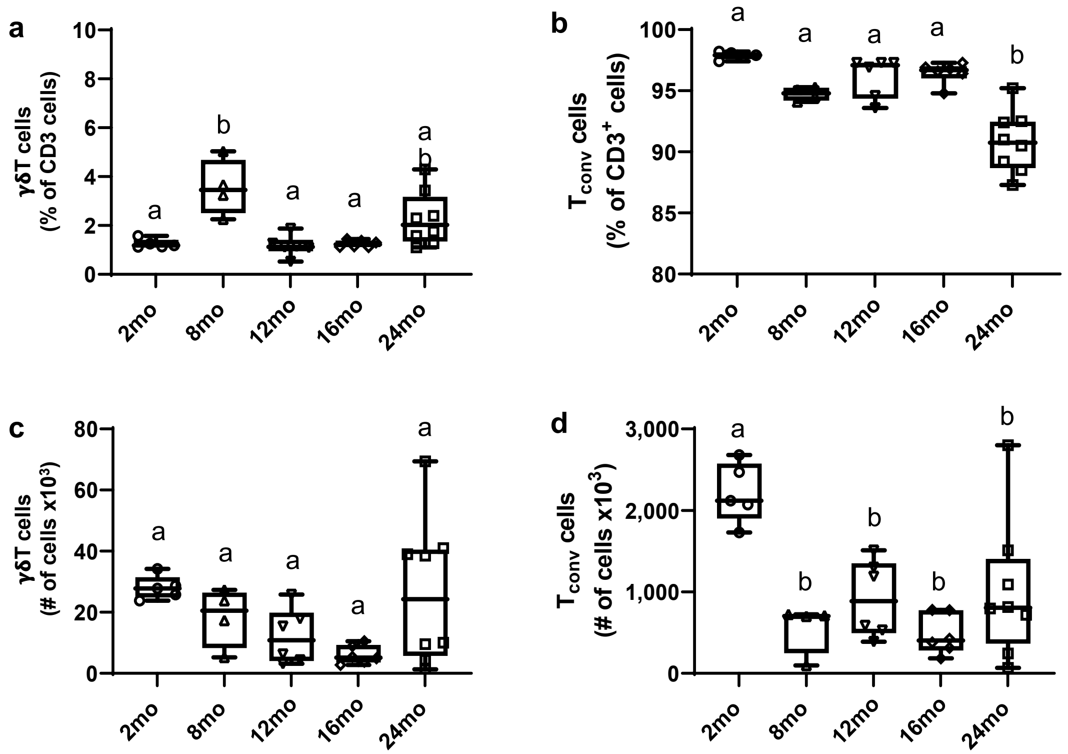


**Supplementary Figure 4. γδ T cells and $T_{conv}$ cells in peripheral lymph nodes over the lifespan. (a-b)** Percentage and **(c-d)** Total number of γδ T and $T_{conv}$ cells were quantified among total CD3+ lymphocytes in lymph nodes (LN) from 2 mo (n=5), 8 mo (n=4), 12 mo (n=6), 16 mo (n=6) and 24 mo (n=8) old male C57BL/6 mice. Data are expressed in box plots from minimum to maximum values with bars representing the mean; each symbol represents an individual mouse. Statistical differences were determined by one-way ANOVA with Tukey's Honest Significant Difference for multiple comparisons. Age groups not connected by the same letters (a,b) are significantly different. mo: month; LN: Lymph node.

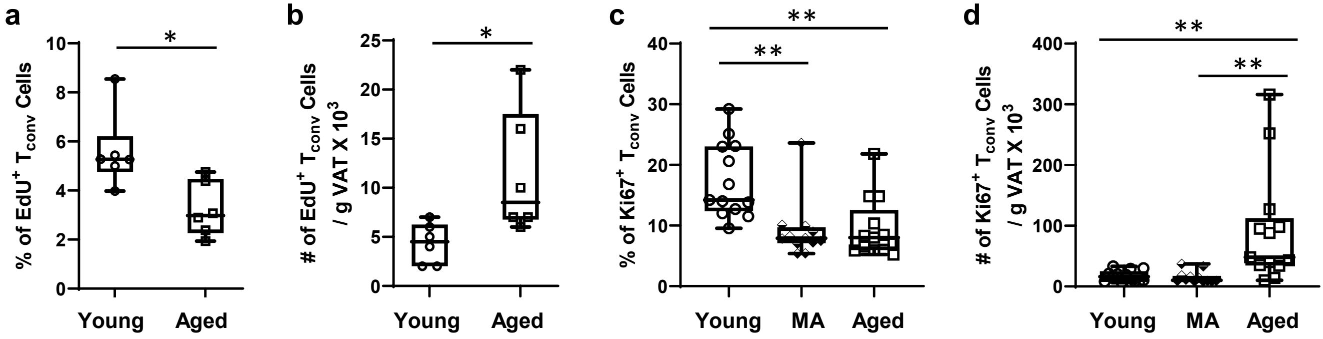


**Supplementary Figure 5. Proliferation among $T_{conv}$ cells in VAT with aging. (a)** Percentage and **(b)** Number of $EdU^{+}$ $T_{conv}$ cells per gram of VAT in young (n=6, 4mo), and aged (n=6, 23mo) male mice. **(c)** Percentage and **(d)** number of $Ki67^{+}$ $\gamma\delta$ T cells per gram of VAT in young (n=13, 4-6mo), middle aged (MA, n=12, 12-16mo) and aged (n=13, 21-25mo) male mice. Data are expressed in box plots from minimum to maximum values with bars representing the mean; each symbol represents an individual mouse. Statistical differences were determined by one-way ANOVA with Tukey's Honest Significant Difference for multiple comparisons. *p<0.05, **p<0.01. EdU: 5-ethynyl-2'-deoxyuridine; VAT: visceral adipose tissue.

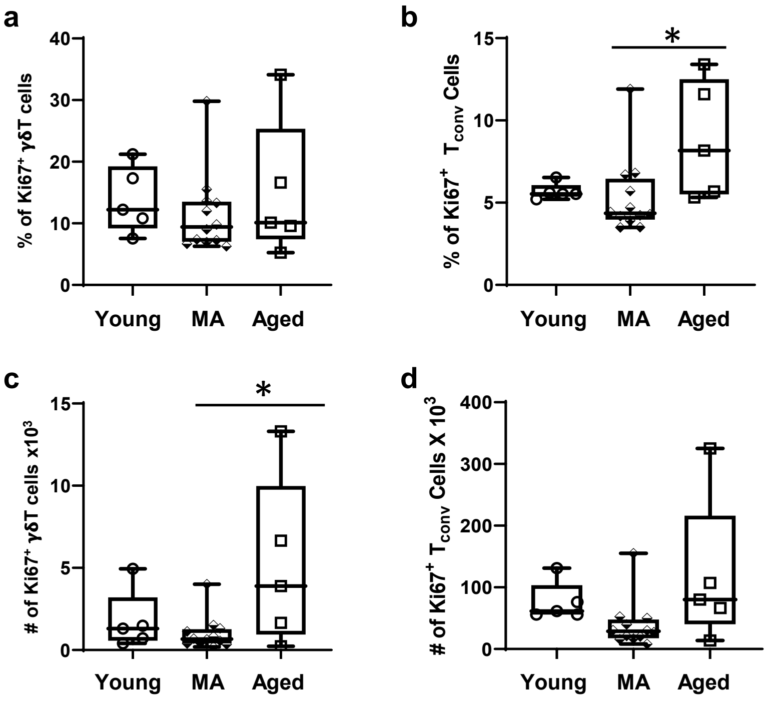


**Supplementary Figure 6. Proliferation among γδ T and $T_{conv}$ cells in lymph node with aging. (a-b)** Percentage and **(c-d)** Number of $Ki67^{+}$ γδ T cells and $T_{conv}$ cells in lymph node (LN) in young (n=5, 4-6mo), middle aged (MA, n=12, 12-16mo) and aged (n=5, 21-25mo) male mice. Data are expressed in box plots from minimum to maximum values with bars representing the mean; each symbol represents an individual mouse. Statistical differences were determined by one-way ANOVA with Tukey's Honest Significant Difference for multiple comparisons. *$p<0.05$. LN: Lymph node.

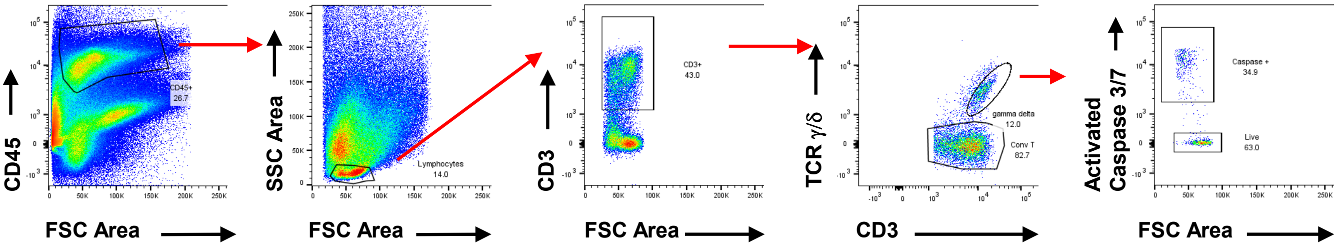


**Supplementary Figure 7.** Representative flow cytometry gating scheme to identify apoptotic cells using CellEvent[TM] Caspase 3/7 Green Flow Cytometry Assay Kit. During apoptosis, caspase-3 and caspase-7 proteins are activated and able to cleave the caspase 3/7 recognition sequence encoded in the kit's dye-conjugated peptide. Cleavage of the recognition sequence and binding of DNA by the reagent labels the apoptotic cells with fluorogenic signal that is captured on the flow cytometer.

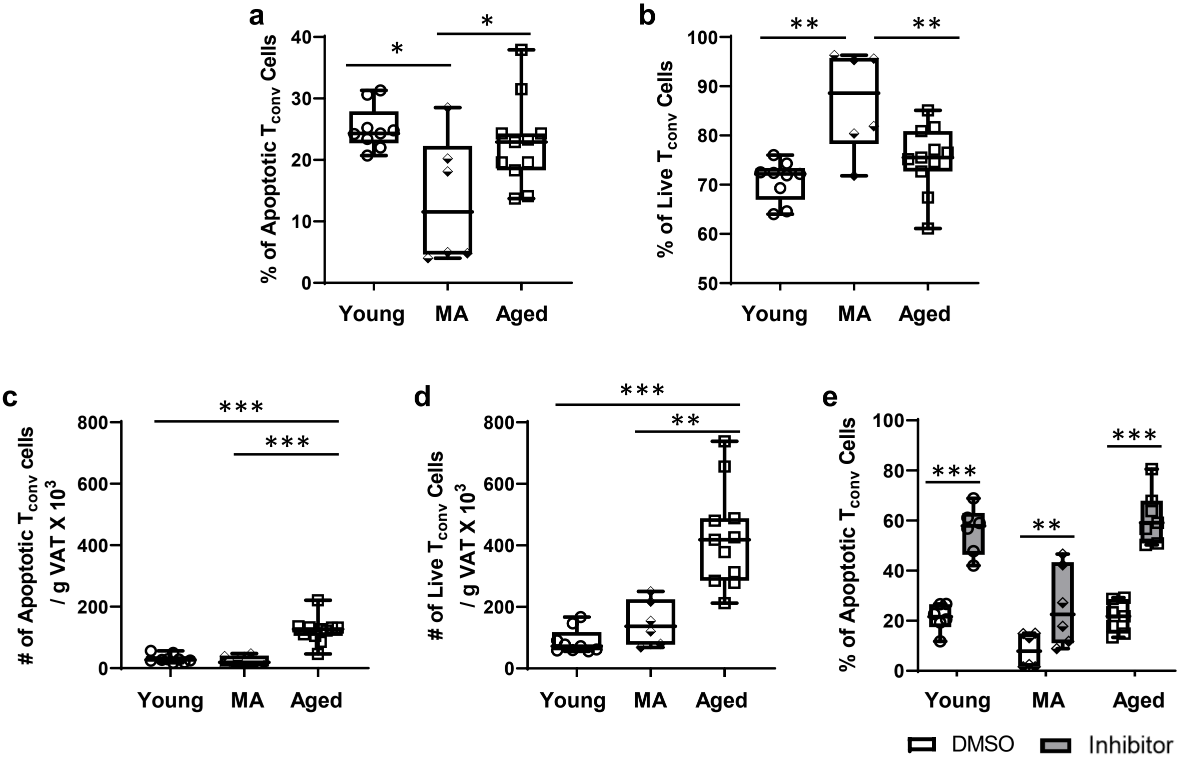


**Supplementary Figure 8. Analysis of apoptosis in VAT-resident $T_{conv}$ cells. (a-b)** Percentage and **(c-d)** Number per gram of VAT of Apoptotic and Live $T_{conv}$ cells in young (n=9, 4mo), middle age (MA, n=6, 12mo) and aged (n=11, 22mo) male mice. Data are expressed in box plots from minimum to maximum values with bars representing the mean; each symbol represents an individual mouse. Statistical differences were determined by one-way ANOVA with Tukey's Honest Significant Difference for multiple comparisons. In cells from separate mice, apoptosis was induced by incubation with Bcl2 family inhibitors ABT737 and Mcl-1 inhibitor II for 3 hours. **(e)** Apoptotic $T_{conv}$ cells in young (n=6, 4mo), middle age (MA, n=6, 12mo) and aged (n=7, 22mo) male mice. Data are expressed in box plots from minimum to maximum values with bars representing the mean; each symbol represents an individual sample. Pairwise statistical differences were detected by paired t-test within each age group, *$p<0.05$, **$p<0.01$, ***$p<0.001$. g: gram; MA: middle-aged; VAT: visceral adipose tissue.

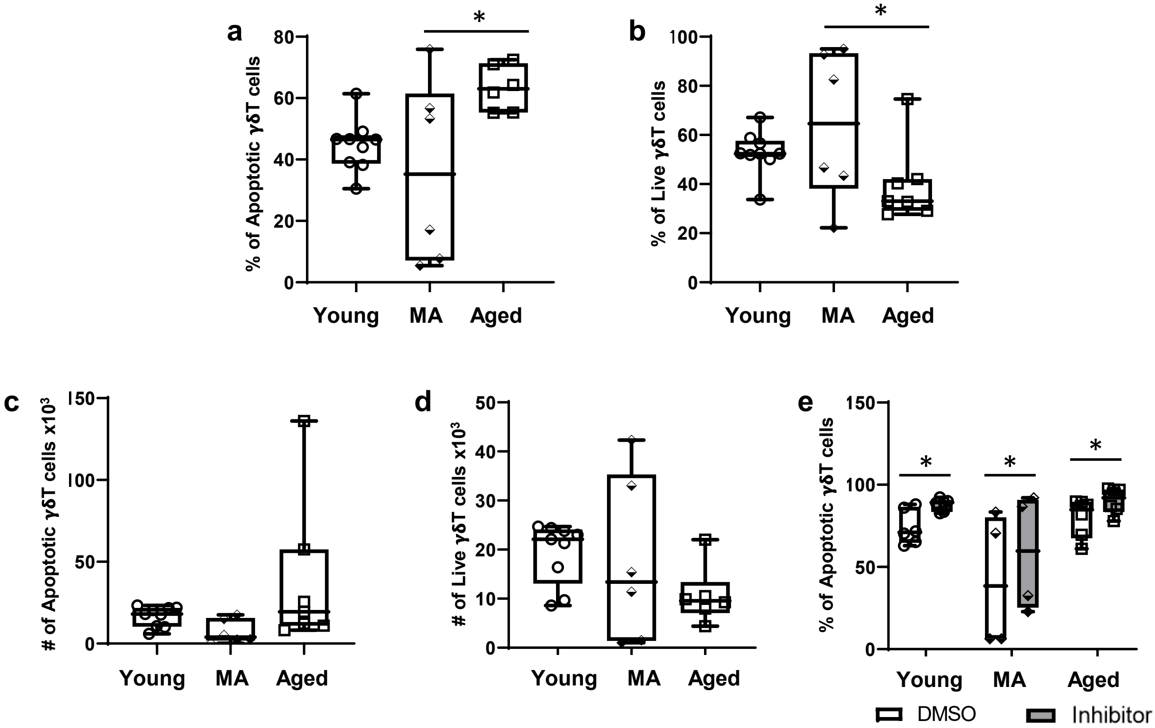


**Supplementary Figure 9. Analysis of γδ T cell apoptosis in peripheral lymph nodes. (a-b)** Percentage and **(c-d)** Number of Apoptotic and Live γδ T cells in LN in young (n=9, 4mo), middle age (MA, n=6, 12mo) and aged (n=11, 22mo) male mice. Data are expressed in box plots from minimum to maximum values with bars representing the mean; each symbol represents an individual mouse. Statistical differences were determined by one-way ANOVA with Tukey's Honest Significant Difference for multiple comparisons. In cells from separate mice, apoptosis was induced by incubation with Bcl2 family inhibitors ABT737 and Mcl-1 inhibitor II for 3 hours. **(e)** Apoptotic γδ T cells in young (n=6, 4mo), middle age (MA, n=6, 12mo) and aged (n=7, 22mo) male mice. Data are expressed in box plots from minimum to maximum values with bars representing the mean; each symbol represents an individual sample. Pairwise statistical differences were detected by paired t-test within each age group, *$p<0.05$. g: gram; LN: Lymph node; MA: middle-aged; VAT: visceral adipose tissue.

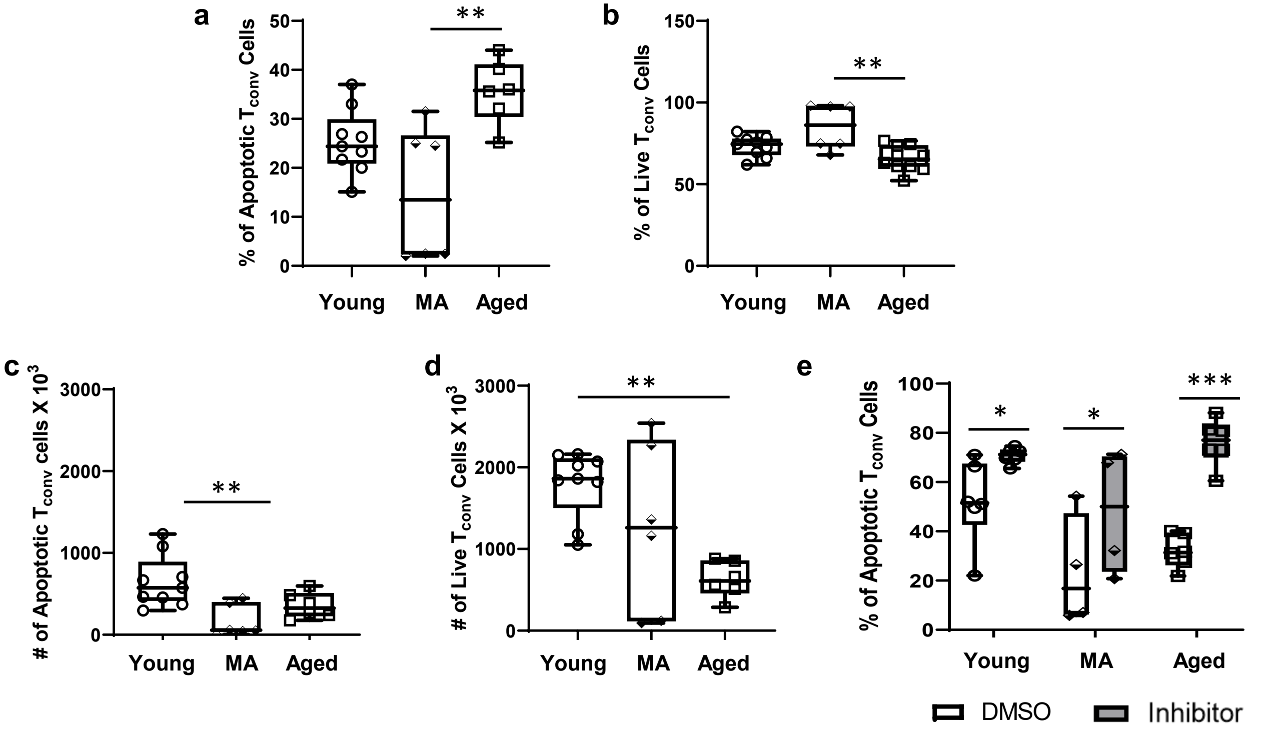


**Supplementary Figure 10. Analysis of $T_{conv}$ cell apoptosis in peripheral lymph nodes. (a-b)** Percentage and **(c-d)** Number per gram of VAT of Apoptotic and Live $T_{conv}$ cells in young (n=9, 4mo), middle age (MA, n=6, 12mo) and aged (n=11, 22mo) male mice. Data are expressed in box plots from minimum to maximum values with bars representing the mean; each symbol represents an individual mouse. Statistical differences were determined by one-way ANOVA with Tukey's Honest Significant Difference for multiple comparisons. In cells from separate mice, apoptosis was induced by incubation with Bcl2 family inhibitors ABT737 and Mcl-1 inhibitor II for 3 hours. **(e)** Apoptotic $T_{conv}$ cells in young (n=6, 4mo), middle age (MA, n=6, 12mo) and aged (n=7, 22mo) male mice. Data are expressed in box plots from minimum to maximum values with bars representing the mean; each symbol represents an individual sample. Pairwise statistical differences were detected by paired t-test within each age group. *p<0.05, **p<0.01, ***p<0.001. g: gram; LN: Lymph node; MA: middle-aged; VAT: visceral adipose tissue.
